# A Novel Questionnaire to Ergonomically Assess Respirators among Health Care Staff: Development and Validation

**Published:** 2018-10

**Authors:** Reza Khani Jazani, Seyed Mohammad Seyedmehdi, Amir Kavousi, Somaye Tahernezhad Javazm

**Affiliations:** 1 School of Health, Safety and Environment, Shahid Beheshti University of Medical Sciences, Tehran, Iran.; 2 Chronic Respiratory Diseases Research Center, National Research Institute of Tuberculosis and Lung Diseases, Shahid Beheshti University of Medical Sciences, Tehran, Iran.

**Keywords:** Ergonomic, N95 respirators, Questionnaire, Confirmatory factor analysis

## Abstract

**Background:**

Health care workers are at a high risk of exposure to infectious diseases spread by airborne transmission. N95 respirators are the most common respirators used in the health care system and negligence in using them may cause health problems. Hence, more emphasis should be on ergonomic aspects of this mask. This study aimed to develop a tool for ergonomic evaluation of these respirators.

**Materials and Methods:**

After reviewing previous studies and employees’ problems in the use of the N95 respirators, 50 questionnaires were designed and their validity was assessed. Then, the questionnaire was completed by 290 staff members of Masih Daneshvari Hospital and its internal consistency and reproducibility were investigated using Cronbach’s alpha coefficient and test-retest method, respectively. Confirmatory factor analysis was used to assess its consistency and internal consistency (construct validity).

**Results:**

With the confirmation of the face and content validities, internal consistency (0.89) calculated by the Cronbach’s alpha coefficient and reproducibility of the questionnaire (0.997; p<0.001) assessed by using the ICC Index, were approved. Following examining internal consistency and stability, the questionnaire convergent construct validity was also confirmed using confirmatory factor analysis.

**Conclusion:**

The questionnaire contained 42 items and it is beneficial to use it in the health care system to evaluate the ergonomic problems of the respirators and to have optimal choice in this respect. Also, it can be used in the promotion of the staffs’ behavior in wearing these respirators when necessary.

## INTRODUCTION

Health care workers are at high risk of exposure to infectious diseases throughout their daily work. The use of personal protective equipment by health care workers is one of the most crucial measures to protect them against diseases. In this regard, the World Health Organization (WHO) and the Center for Disease Control and Prevention (CDC) proposed the N95 respirator masks in order to prevent the spread of infection through the air (1, 2). The N95 respirator masks are compressed facial masks, so that they should be fixed on the face to provide respiratory protection (3). A significant concern in the use of these masks is their reliability to impede the contaminant transport to an individual’s breathing tract. This depends upon the degree of adaptation and appropriateness of the respirator mask for the mask wearer (4).

Although great efforts have been made to enhance the potentials of respirators, few studies have investigated the ergonomic aspects of using masks and the relationship between respiratory masks, human beings, and environment (5). However, taking the principles of ergonomics into account to design a device is of paramount importance and can be considered as a critical factor affecting its success (6). Knowledge of ergonomics can be used for designing, constructing, and selecting a device through measuring its compliance with the user. Given that the respirators protect human beings; it is necessary to put more emphasis on ergonomic aspects of the respirator masks.

Over the recent decades, a lot of studies have been carried out to determine the limitations of the respirators with respects to Human Factors (HF) and to resolve them. Several studies conducted on respirator masks in the field of the ergonomics have mainly focused on the respirator’s fit, their comfort, their impact on the performance of individuals wearing them, and the mood of an individual while using the respirators.

To improve the respiratory masks user satisfaction, researchers have seeked to eliminate the respirators’ poor face-mask compliance. Three features of the masks, which are closely related to the extent of compliance, include: respirator size, respirator shape and respirator edges. The face-mask compliance is also related with the size, shape and roughness of an individuals’ face (7).

According to a study conducted by Grinshpun et al., seal between face **and** respirator **is th**e most important part responsible for any leakage of contaminants (8). Following the issue of respirator fit, the respirator comfort was the most significant point considered by many researchers. In this regard, poor fit and discomfort were the most common reasons cited for not wearing a mask (7).

Comfort should be an important selection criterion because when a person is comfortable using a respirator, he would probably wear it more accurately. Respirator masks may impose physiological stress on mask wearers. Comfort of the respirator masks is usually determined based on a combination of physical and physiological factors (9). It can be measured objectively or subjectively. Different aspects of comfort include the impact of respirators on humidity and heat inside the mask (an individual’s breathing zone) and pressure imposed on an individual’s face, nose and chin as well as the cardiac and pulmonary effects measured subjectively or objectively.

The third issue raised in the field of ergonomics for the respirator masks is their impacts on the performance of individuals wearing them. Previous studies have shown that the respirator masks can prevent the mask wearers’ performance. Some studies have also proposed that the mask wearers’ performance is decreased as the performance of the respirator masks increases. Some studies have investigated the effects of the respirator masks on tasks such as walking, running, acting, and so on. Their effect on vision, hearing and physical, cognitive and psychomotor activities is also researched. A study showed that the respirators with higher levels of protection, lower physiological and psychological abilities of mask wearer, especially if the wearer is performing physical works (10).

The fourth issue associated with the respirator masks in the field of ergonomics is an individual’s mental state while wearing masks. Its impact on a person’s psychological mood or anxiety is a vital factor to evaluate and approve a new respirator mask (11). During the recent years, there has been an increasing interest by the designers to introduce research methods in the field of ergonomics in order to assess and translate the respirator masks from a human perspective.

Since no study has been conducted to provide a complete evaluation of ergonomic aspects of a half-face respirator and checklists and questionnaires are the most convenient and common methods being used to investigate the ergonomic aspects of these devices, the present study aimed to introduce designers and users the N95 Respirators a novel questionnaire to ergonomically assess respirators among health care staff as an effective instrument to evaluate and compare ergonomic aspects of the N95 respirator masks used in the health care system.

## MATERIALS AND METHODS

This study was a descriptive-analytic study. In this study, the content validity, Cronbach’s alpha coefficient and test-retest methods were used to evaluate the validity, internal consistency and reliability of the designed questionnaire “N95 Respirator Masks Ergonomic Analysis among Medical Staff”. Confirmatory factor analysis was used to assess its consistency and internal consistency (construct validity). The following procedures were adopted:

### 1) Developing the questionnaire:

The first step was to determine the questionnaire content domain. The content validity of the N95 Respirator Masks Ergonomic Analysis among medical staff was specified after reviewing previous studies obtained from scientific resources in the field of the respirator masks ergonomics (7, 9). The ergonomic aspects of the N95 respirator masks were determined after studying and analyzing the factors (i.e. groups of descriptors) underlying the mask fit to user’s face and their ease of use and investigating studies conducted on the impact of respirator masks on individuals’ performance and according to the results of several studies indicating the effect of respirator masks on individuals’ psychological condition (10–13). Consequently, some items covering these dimensions of the N95 respirator mask for health care personnel were designed.

The questionnaire consists of four major dimensions: respirator fit, comfort, their impact on the performance of individuals wearing them, and the mood of an individual while using the respirators (13). There were also some items included to gather information about the respondent’s demographic information, awareness, received training, attitudes, and the frequency of wearing masks in order to further identify and specify the audience. Then, Masih Daneshvari Hospital was selected as research setting since it is the national center for TB patients and its personnel are at high risk. Thus, the researchers personally attended in the research setting for several sessions and developed the items with regard to the above mentioned criteria. The first draft of the questionnaire was prepared, which contained 50 five-point Likert scale questions.

### Identifying panel members to determine validity:

At this stage, the panel members were identified. To make an accurate judgment, the panel members were selected from experts active in the field of the questionnaire content domain. In some studies, the minimum number of panel members is suggested to be four persons (14, 15). In this study, eight experts participated in the validation process to achieve greater consensus with high level of confidence. Eight experts were selected and the researchers met them in person. Members of content validity were experts in ergonomics, occupational medicine and Occupational Health Engineering.

### 2) Validity and Reliability

#### Determining Face Validity

The first step to determine validity is to specify the face validity since if a change is required, no problem occurs for the entire questionnaire (16). At this stage, the panel members’ comments were applied and the researchers attempted to make the question wordings clear, fluent and appropriate.

#### Determining Content Validity

In order to determine the questionnaire content validity, the questionnaires were submitted to the panel members once again and they asked to select one of three options (namely necessary, useful but not necessary, and not necessary) for each item. They were also asked to provide further comments on items considered as necessary or not necessary.

In order to determine the content validity, the panel members’ comments being assigned based on necessity were quantified using the Content Validity Ratio (CVR).

Given the number of panel members (n=8) who participated in the validation process, the minimum CVR value accepted for each question was considered to be equal to 0.75% (17) and items with smaller VCR values were excluded.

#### Determining Content Validity Index

Content Validity Index (CVI) CVI is equal to the CVR mean of valid items. It represents integrity of judgments about the validity or enforceability of the final tool.

#### Internal consistency and reproducibility of the final questionnaire

The term reliability generally refers to the consistency of a measurement specified by a tool at same conditions (18, 19). Reliability coefficient ranges from zero to one. Reliability coefficients of zero and one represent lack of consistency and perfect reliability, respectively (20–22). At this point, 290 questionnaires were completed by the Masih Daneshvari Hospital medical staff. Then, the questionnaire internal consistency was determined using Cronbach’s alpha coefficient.

In order to test the questionnaire reproducibility, the questionnaire was resubmitted after two weeks to 45 individuals who had previously completed the questionnaires to examine the questionnaire reproducibility using test-retest method and intra-class correlation coefficient index.

#### Validity

After investigating the internal consistency of the questionnaire items, confirmatory factor analysis was used to assess the relationship between variables and identify the factors (23).

## RESULTS

After developing the first draft of the **“**N95 Respirator Masks Ergonomic Analysis among Medical Staff Inventory” with 50 items and confirming the face validity of the questionnaire, the content validity ratio of 45 questions was acceptable (CVR> 0.75) and they were included in the final questionnaire. The index was statistically lower than the acceptable value for the five other questions so that they were excluded from the final questionnaire. The validity index value (=0.9) for the remaining 45 questions was acceptable.

When the questionnaires were completed by 290 medical staff, Cronbach’s alpha coefficients were determined for different sections of the questionnaire (Table 1). The values presented for each aspect and the entire questionnaire were greater than 0.8, indicating high reliability of the questionnaire.

**Table 1.** Relevance and reliability of the questionnaire data

Dimension	KMO index	Bartlett	Sig.	Cronbach’s alpha
Training	0.83	2198.6	<0.001	0.86
Comfort	0.85	1458.4	<0.001	0.88
Human performance	0.77	482.3	<0.001	0.77
Mood	0.82	981.5	<0.001	0.86
Entire questionnaire	0.84	4995.6	<0.001	0.89

Reproducibility of the questionnaire was assessed by using the ICC Index and test-retest method (ICC=0.997; P<0.001), which indicates that it is highly reproducible.

In order to verify this claim that the questionnaire consists of four major dimensions (namely Face-mask compliance, ease of use, their impact on the performance of individuals wearing them, and the mental state of an individual while using the respirators), factor analysis was used. Moreover, the Bartlett and KMO Index were used to check the suitability of data for factor analysis. As shown in table 1, the KMO index is acceptable for all aspects of the questionnaire and Bartlett value is significant, indicating the suitability of data to be used for factor analysis.

In the primary model, the goodness of fit values was not at an acceptable level. A better model was obtained after removing and mitigating items whose impact was not significant or had standardized coefficients less than 0.5. In this model, all indices were highly satisfactory and all conditions of internal consistency or convergent construct validity were observed for all components (Table 2).

**Table 2.** Standardized coefficients of confirmatory factor analysis in modified model to study the impact of each item on various aspects of the questionnaire

Items	Standardized coefficients	Standard error	Sig.	Items	Standardized coefficients	Standard error	Sig.
**The effect of items on training**				**The effect of items on ease of use**			
1	0.723	0.066	<0.001	23	0.639	0.074	<0.001
2	0.784	0.047	<0.001	24	0.605	0.079	<0.001
3	0.870	0.093	<0.001	25	0.745	0.081	<0.001
4	Removed			26	0.780	0.077	<0.001
5	0.743	.095	<0.001	27	0.815	0.080	<0.001
6	0.616	.086	<0.001	28	0.526	0.060	<0.001
7	0.580	0.092	<0.001	29	0.633	0.078	<0.001
8	0.723	0.078	<0.001	30	0.501	0.101	<0.001
9	Removed			31	Removed		
10	0.387	0.068	<0.001	32	0.706	0.062	<0.001
11	Removed			-	-	-	-
12	Removed			-	-	-	-
**The effect of items on performance**				**The effect of items on mental state**			
33	Removed			38	0.790	0.082	<0.001
34	0.774	0.071	<0.001	39	0.870	0.059	<0.001
35	0.866	0.062	<0.001	40	0.804	0.068	<0.001
36	0.592	0.065	<0.001	41	0.857	0.064	<0.001
37	0.723	0.074	<0.001	42	0.628	0.072	<0.001

Tables 3 and 4 are goodness of fit tools for the revised conceptual model. In examining the validity of the questionnaire using confirmatory factor analysis, the revised measurement model indices showed the model acceptable fit.

**Table 3.** The accuracy of the questionnaire revised conceptual model

Index	acceptable value	estimated value of the modified model
CMIN/DF	<3	2.15
GFI	>0 .8	0.866
AGFI	>0 .8	0.830
NFI	>0.9	0.896
CFI	>0 .9	0.934
TLI	>0 .9	0.922
IFI	>0 .9	0.934
RMSEA	<0.8	0.063
PNFI	>0 .5	0.754

**Table 4.** Component of internal consistency

	ASV	MSV	AVE	CR
Training	0.016	0.038	0.503	0.886
Performance	0.277	0.471	0.556	0.831
Mental	0.212	0.471	0.631	0.894
Ease of use	0.185	0.353	0.496	0.876

Internal consistency conditions were also observed for all components. This means that all coefficient values are significant and greater than 0.5. According to table 4, the AVE values are greater than 0.5 and less than CR for all components.

Since the CR values are greater than 0.7, the combined reliability is also achieved. Since the AVE values are greater than the MSV and ASV values for all components, divergent validity is also established for all components.

## DISCUSSION

After confirming the face and content validities, a 45-item questionnaire was developed. Cronbach’s alpha values obtained for awareness and training, comfort, human performance and mood dimensions were acceptable and equal to 0.86, 0.88, 0.77, and 0.86, respectively. The Cronbach’s alpha value for the entire questionnaire (0.89) was also acceptable and significant. Reproducibility of the questionnaire (0.997; P<.001) was assessed by using the ICC Index, which indicates that it is highly reproducible.

Factor analysis revealed that a number of questions should be removed in order for the questionnaire to be valid. According to the experts in the field, some of the questions (two questions from training dimension on the mask user familiarity with the user seal check and fit test and a question from performance dimension on the effect of mask on the performance of mask wearers performing physical tasks) could not be removed since they regarded the lack of agreement of these questions with other questions related to those aspects associated with training problems in proper use of masks and type of activity among the study population. Furthermore, the dimension face-mask compliance was not included in the factor analysis due to differences in responses (responses were provided in two forms of yes/No or fill-in-the-blanks). Finally, a 45-item questionnaire containing five dimensions of awareness and training, comfort, performance, mood and fit was prepared.

Given that the correct choice of respiratory protective equipment regardless of the specifications and limitations of the users reduces their efficiency and can lead to irreparable damages to medical staff and hospital infection control system, the “N95 Respirator Masks Ergonomic Analysis among Medical Staff Inventory” can present a full assessment of ergonomic aspects of a half-face respirator (24, 25). It not only can help the mask designers to increase the quality of the product but also can be used by the respiratory protection program executors at the hospital in order to enable them to select the correct type of respirator.

It can of great contribution in monitoring this program (26, 27). The questionnaire contained 42 items and it is beneficial to use it in the health care system to evaluate the ergonomic problems of the masks and to have optimal choice in this respect and it can be used in the promotion of the staffs’ behavior in wearing these masks when necessary.
